# Clinical Evaluation of Postoperative Day 3 Serum Lipase in Relation to Clinically Relevant Pancreatic Fistula After Distal Pancreatectomy: A Retrospective Cohort Study

**DOI:** 10.3390/biomedicines14092020

**Published:** 2026-09-08

**Authors:** Oliwia Grząsiak-Kraj, Tomasz Kraj, Krzysztof Poznański, Agata Grochowska, Piotr Hogendorf, Adam Durczyński, Janusz Strzelczyk

**Affiliations:** 1 Department of General and Transplant Surgery, Norbert Barlicki Memorial Teaching Hospital No. 1, Medical University of Lodz, 22 Kopcinskiego Street, 90-153 Lodz, Poland; 2 Department of Vascular Surgery and Angiology, Ministry of the Interior and Administration Hospital, 90-153 Lodz, Poland; 3 Institute of Medical Expertises in Lodz, 90-153 Lodz, Poland

**Keywords:** distal pancreatectomy, postoperative pancreatic fistula, serum lipase, postoperative complications, biomarker, risk stratification, postpancreatectomy acute pancreatitis

## Abstract

**Background/Objectives:** Clinically relevant postoperative pancreatic fistula (CR-POPF) remains a major complication after distal pancreatectomy. Postoperative pancreatic enzyme elevation is increasingly recognized as a marker of pancreatic remnant injury, but serum lipase after distal pancreatectomy has been less well characterized than serum amylase and drain-fluid markers. This study evaluated postoperative day 3 (POD3) serum lipase as a routine biochemical marker associated with CR-POPF. **Methods:** We retrospectively analyzed 202 consecutive adults who underwent distal pancreatectomy between 2016 and 2026. CR-POPF was defined as International Study Group on Pancreatic Surgery grade B or C. POD3 lipase was available in 157 patients, including 17 CR-POPF events. **Results:** POD3 lipase was higher in patients with CR-POPF than in those without it (median 74 vs. 31 U/L; *p* < 0.001). Each doubling of lipase was associated with higher odds of CR-POPF (OR 2.59, 95% CI 1.54–4.37). The AUC was 0.79 (95% CI 0.67–0.90) and remained 0.79 after bootstrap optimism correction. At 64 U/L, the negative predictive value was 96%; at 3 × ULN, specificity was 99%. **Conclusions:** POD3 serum lipase provides a readily available serum biochemical marker, not requiring drain-fluid sampling, associated with CR-POPF after distal pancreatectomy. The findings are exploratory and require external validation before clinical implementation.

## 1. Introduction

Clinically relevant postoperative pancreatic fistula (CR-POPF) remains one of the most important pancreas-specific complications after distal pancreatectomy [1]. Although postpancreatectomy mortality is now defined using a standardized ISGPS classification [2], CR-POPF continues to drive postoperative morbidity, delayed recovery, prolonged drainage, percutaneous or endoscopic intervention, readmission, and longer hospital stay [3]. The 2016 International Study Group on Pancreatic Surgery (ISGPS) update refined the definition of postoperative pancreatic fistula by distinguishing biochemical leak from clinically relevant grade B and C fistulas [4,5], thereby focusing attention on complications that alter postoperative management.

Distal pancreatectomy represents a distinct anatomical and pathophysiological setting compared with pancreatoduodenectomy [6]. After pancreatoduodenectomy, fistula formation is related to failure of a pancreaticoenteric anastomosis, whereas after distal pancreatectomy it arises from the transected pancreatic remnant [6]. Established risk factors for CR-POPF after distal pancreatectomy include pancreatic thickness, duct diameter, body mass index, and technical factors related to stump closure [7,8]. The role of pancreatic texture, well established after pancreatoduodenectomy, appears less consistent after distal pancreatectomy [9]. These factors have been incorporated into preoperative and intraoperative risk models [10,11,12,13], but such tools are fixed at the time of surgery and do not capture the evolving postoperative biological response of the remnant.

Postoperative biochemical markers can provide dynamic information during the early postoperative course. Drain-fluid amylase remains the reference biochemical test for identifying pancreatic leakage, but it requires a drain to be present and a dedicated sample to be obtained [14]. Selective drain placement and early drain removal are increasingly considered after pancreatic surgery [15], while drain-fluid interpretation may also be affected by contamination or colonization [16]. A routine serum biomarker could therefore complement existing risk assessment without requiring drain-fluid sampling.

Post-pancreatectomy acute pancreatitis and postoperative pancreatic enzyme elevation have become increasingly relevant to the biology of pancreas-specific complications [17,18,19,20,21,22,23]. Importantly, a large European multicenter cohort of 1192 distal pancreatectomies demonstrated that postoperative hyperamylasemia was associated with both the occurrence and severity of POPF [24]. Prior studies have also shown clinically relevant associations between postoperative pancreatic enzymes and fistula after pancreatic resection, particularly after pancreatoduodenectomy and in mixed resection cohorts [25,26,27,28,29,30,31]. These studies establish a broader relationship between postoperative acinar injury and fistula; however, the clinical behavior of serum lipase specifically on POD3 after distal pancreatectomy remains less well characterized than serum amylase or drain-based markers.

POD3 is a clinically relevant window because transient perioperative enzyme elevation may have begun to resolve, whereas persistent or newly elevated serum lipase may reflect ongoing parenchymal or stump inflammation or an evolving leak [32]. This time point also coincides with common decisions regarding drain management, imaging, monitoring intensity, and discharge planning [33]. The present study therefore evaluated the association and discriminatory performance of POD3 serum lipase in relation to CR-POPF after distal pancreatectomy, examined clinically interpretable thresholds and the relationship with ISGPS severity, and compared lipase with other routinely available laboratory markers. The aim was clinical characterization of a simple postoperative biomarker rather than derivation of a stand-alone prediction rule.

## 2. Materials and Methods

### 2.1. Study Design, Ethics, and Reporting

This single-center retrospective cohort study included consecutive adult patients who underwent distal pancreatectomy at the Department of General and Transplant Surgery, Medical University of Lodz, between 1 January 2016 and 1 May 2026. The study was conducted in accordance with the Declaration of Helsinki and relevant institutional guidelines and was approved by the Bioethics Committee of the Medical University of Lodz (approval no. RNN/133/26/KE/KE, 12 May 2026). The committee waived the requirement for individual informed consent because the study was retrospective and used anonymized, routinely collected clinical data. Reporting follows the STROBE statement for cohort studies [34]; the completed checklist is provided as Supplementary File S1.

### 2.2. Patient Population and Follow-Up

Patients were eligible irrespective of final histopathological diagnosis, provided that distal pancreatectomy was performed. Distal pancreatectomy with or without splenectomy was included. Extended multivisceral resections were not excluded if they were performed for locally advanced disease with the intention of achieving an R0 resection. No trauma-related distal pancreatectomies were included. Patients were identified from institutional operative records and hospital documentation. Exclusion criteria were pancreaticoduodenectomy, total pancreatectomy, central pancreatectomy, pancreatic enucleation, procedures not involving distal pancreatic transection, and insufficient postoperative documentation to determine the occurrence or absence of CR-POPF. When more than one pancreatic resection was performed in the same patient during the study period, only the first eligible distal pancreatectomy was included. Postoperative outcomes were assessed during the index hospital stay and documented readmissions or follow-up visits within 30 days after surgery.

### 2.3. Postoperative Management and Data Collection

In the study center, intra-abdominal drains are routinely placed after distal pancreatectomy. Drain removal is performed as early as clinically appropriate and is guided by postoperative condition, drain output volume and appearance, biochemical findings, and the presence or suspicion of pancreatic fistula. Because of the retrospective design, postoperative management was not protocolized for this study and reflected routine clinical practice during the study period. Perioperative and postoperative data were extracted from operative reports, anesthesia records, laboratory databases, discharge summaries, and follow-up documentation. Collected variables included demographic characteristics, operative details, histopathological diagnosis, postoperative laboratory parameters, drain management, postoperative complications, need for interventional drainage or reoperation, length of hospital stay, readmission, and mortality. Serum lipase values were recorded for POD1 and POD3. Only measurements drawn exactly on POD3 were used for the primary marker analysis. Lipase was measured by enzymatic colorimetric assay in the hospital central biochemistry laboratory; the institutional upper limit of normal (ULN) remained 67 U/L throughout the study period. Peak serum lipase and peak serum amylase (POD1-3 and POD1-7), C-reactive protein (CRP), full blood count, and selected preoperative parameters were also recorded.

### 2.4. Definition of Postoperative Pancreatic Fistula

Postoperative pancreatic fistula was graded according to the 2016 ISGPS definition [4]. Biochemical leak was not included in the primary endpoint. Grade B and C fistulas were classified as CR-POPF; biochemical leak and absence of leak were both classified as no CR-POPF for the primary binary analysis. Grades were assigned retrospectively by the study team from complete clinical records, including laboratory data, against the 2016 ISGPS criteria; assessors were not blinded to laboratory results.

### 2.5. Laboratory Markers

The primary marker, specified before the statistical analyses for this study, was serum lipase concentration on POD3. Serum lipase was analyzed as a continuous variable and at clinically interpretable thresholds, including the institutional ULN and three times the ULN. POD1 serum lipase was used in a secondary analysis to assess whether the POD3 association persisted in patients without an early postoperative lipase surge; an early lipase surge was defined a priori as POD1 lipase >3 × ULN (201 U/L), and classification required an available POD1 measurement. Other routinely available postoperative laboratory parameters, including peak serum lipase, peak serum amylase, and CRP, were evaluated as secondary or exploratory markers.

### 2.6. Statistical Analysis

No formal sample-size calculation was performed because the retrospective cohort included all eligible distal pancreatectomies during the study period. Analyses followed a complete-case approach: missing laboratory data were not imputed, and each analysis was restricted to patients with an available measurement for the relevant variable. Availability of the primary marker was compared with CR-POPF status using Fisher’s exact test. Because several laboratory variables were right-skewed and the event group was small, continuous variables were summarized as medians with interquartile ranges and compared using the Mann–Whitney U test; categorical variables were compared using Fisher’s exact test. Serum lipase on POD3 was log2-transformed for regression analysis so that the odds ratio (OR) represented the change in odds of CR-POPF per doubling of concentration. Univariable logistic regression was used to estimate the marker-outcome association. Given the 17 events in the primary analytic sample and incomplete availability of established confounders, no multivariable prediction model was fitted; the analysis was intentionally limited to a specified single-marker association and internal validation. Discrimination was quantified by the area under the receiver operating characteristic curve (AUC), with 95% confidence intervals calculated by the DeLong method. Bootstrap internal validation with 2000 replicates was used to estimate optimism in the AUC and in performance at the data-derived Youden threshold. In each resample, the ROC model and the Youden threshold were re-derived, performance was evaluated in the resample and in the original data, and the mean difference (optimism) was subtracted from the apparent estimates; this constitutes internal validation only. Sensitivity, specificity, positive predictive value (PPV), and negative predictive value (NPV) were calculated at the institutional ULN, the Youden-optimal threshold, and 3 × ULN. Positive and negative likelihood ratios were additionally calculated at each threshold. The ordered association across ISGPS severity categories was assessed using the Jonckheere-Terpstra trend test and supplemented by the Kruskal–Wallis test. The severity analysis was prespecified in two forms: an inferential analysis with grades B and C combined into the clinically relevant category, and a descriptive four-grade display. Predictors of availability of the primary marker were explored by logistic regression (age, sex, operative approach, calendar year, length of stay), and results were additionally examined by study era (2016–2020 versus 2021–2026). For each CR-POPF case, the postoperative day of the first drain-fluid amylase exceeding three times the serum ULN was determined. For the family of ten laboratory markers, multiplicity was controlled using the Benjamini–Hochberg false discovery rate procedure; raw *p*-values and adjusted q-values are reported. All tests were two-sided, and *p* < 0.05 was considered statistically significant. Analyses were performed in R version 4.3 (R Foundation for Statistical Computing, Vienna, Austria) using the pROC, PMCMRplus, dplyr, and ggplot2 packages.

## 3. Results

### 3.1. Study Population

A total of 202 consecutive patients who underwent distal pancreatectomy during the study period were included in the analysis. Median age was 65 years (IQR 57–72; range 22–85), and 133 patients (66%) were women. Fifty patients (25%) underwent laparoscopic distal pancreatectomy, whereas the remaining patients underwent open surgery; median operative time was 85 min (IQR 65–120). Median length of hospital stay was 8 days (IQR 7–12). Histopathological diagnosis was available in 137 patients. The most frequent diagnoses were pancreatic neuroendocrine tumor and pancreatic ductal adenocarcinoma, each recorded in 26 patients, followed by intraductal papillary mucinous neoplasm in 19 patients, serous cystadenoma in 19 patients, and mucinous cystic neoplasm in 11 patients. The remaining reports comprised non-neoplastic lesions (*n* = 23), other benign lesions (*n* = 7), pancreatic metastases (*n* = 3), other malignant tumors (*n* = 2), and a solid pseudopapillary neoplasm (*n* = 1). A malignant diagnosis was present in 41 patients. Documented hypertension was present in 77 patients (38%), and documented diabetes in 51 patients (25%), although comorbidity data were incomplete. Clinically relevant postoperative pancreatic fistula occurred in 20 patients (9.9%): grade B in 16 patients and grade C in 4. Compared with patients without CR-POPF, those who developed CR-POPF were older (median 70 versus 65 years; *p* = 0.025) and had a longer hospital stay (median 19 versus 8 days; *p* < 0.001); grade C fistulas, although few in number, were associated with a median stay of 42 days. Management of the 20 CR-POPF cases was as follows: among grade B fistulas (*n* = 16), five patients underwent endoscopic intervention (ERCP with pancreatic duct stenting or sphincterotomy), two underwent percutaneous ultrasound-guided drainage of a peripancreatic collection, two received somatostatin analogs, one required total parenteral nutrition, at least four received targeted antibiotic therapy, and six were discharged with the drain in situ or required prolonged drainage, the remainder being managed conservatively; among grade C fistulas (*n* = 4), three patients required relaparotomy for pancreatic stump necrosis or stump bleeding (one patient twice), three required intensive care, one required continuous renal replacement therapy, and two died. There were borderline differences in documented hypertension (60% versus 36%; *p* = 0.050) and laparoscopic approach (45% versus 23%; *p* = 0.052). No significant differences were observed for sex, operative time, diabetes, or malignancy status. Patient characteristics and postoperative outcomes stratified by CR-POPF are shown in Table 1.

### 3.2. Availability of POD3 Serum Lipase

Serum lipase on postoperative day 3, the prespecified primary marker, was available in 157 patients (78%), including 17 of the 20 patients who developed CR-POPF; the primary discrimination analysis was therefore based on 157 patients and 17 events. Among the three patients with CR-POPF without an available POD3 lipase measurement, one had a grade B and two had grade C fistulas (two of the four grade C cases overall); review of the source records indicated fulminant early courses in the two grade C cases (one patient died on POD3 and one required early reoperation and intensive care), whereas no reason could be established for the grade B case. Availability of a POD3 lipase measurement was not significantly associated with the outcome: CR-POPF occurred in 17 of 157 patients (10.8%) with an available measurement and in 3 of 45 (6.7%) without one (*p* = 0.574, Fisher’s exact test). This comparison has limited power and does not by itself establish that the missing-data mechanism is ignorable. In a logistic model of lipase availability (age, sex, operative approach, calendar year, length of stay), the only significant predictor of availability was calendar year (OR 1.23 per year, 95% CI 1.09 to 1.40; *p* = 0.001), with availability rising from 57% in 2016–2020 to 82% in 2021–2026, consistent with a substantial institutional (temporal) component of missingness (Supplementary Table S1). Severity-related informative missingness nevertheless remains plausible, as two of the four grade C fistulas lacked POD3 measurements because of fulminant postoperative courses; any apparent relation between availability and length of stay should not be interpreted causally, because length of stay accrues after the POD3 sampling time point.

### 3.3. Association of POD3 Serum Lipase with CR-POPF

**Figure 1:**
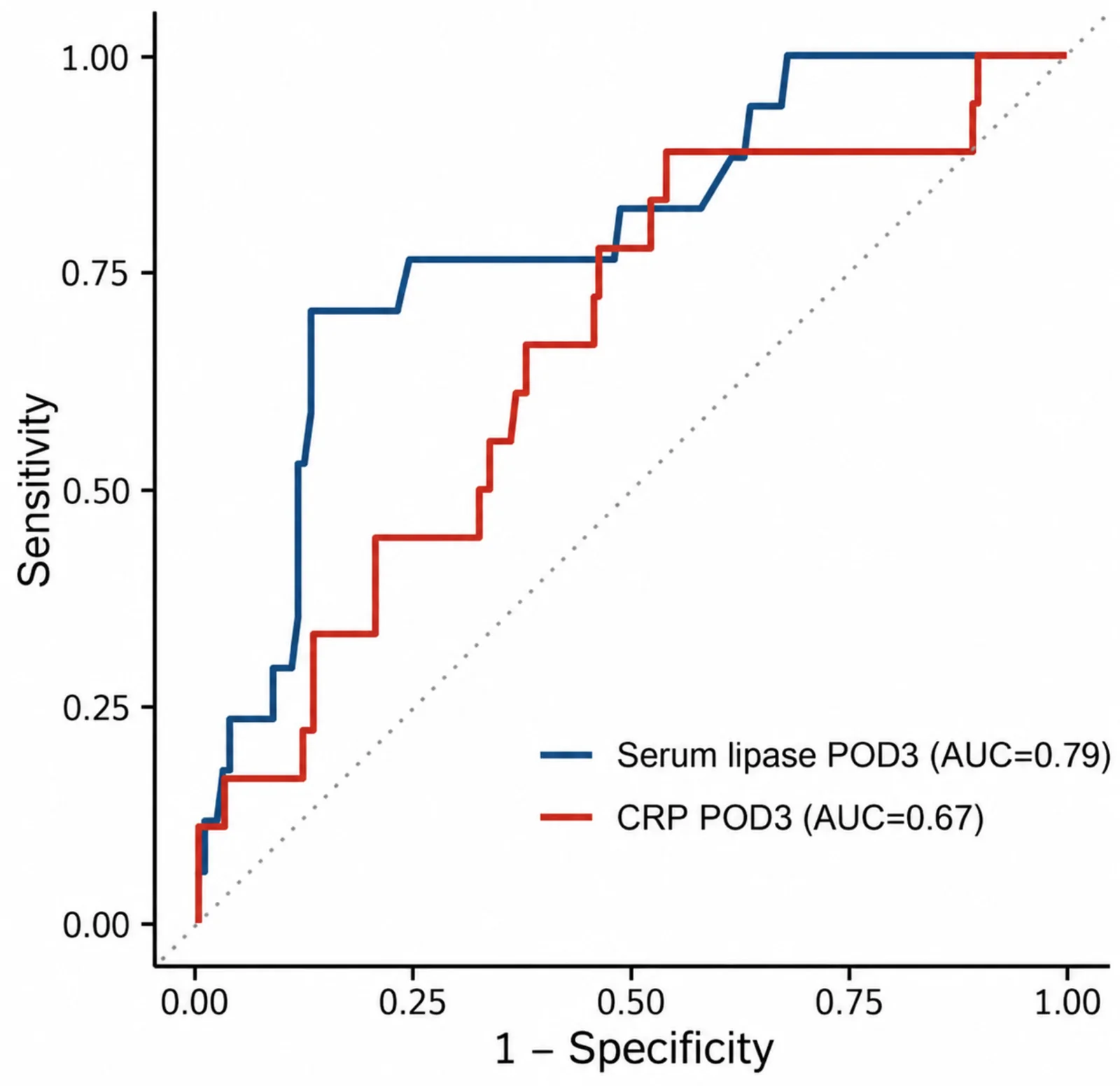
Receiver operating characteristic curves for postoperative day 3 (POD3) serum lipase and C-reactive protein (CRP) as markers of clinically relevant postoperative pancreatic fistula (CR-POPF). Curves use all available observations for each marker (lipase, *n* = 157; CRP, *n* = 185). AUC, area under the receiver operating characteristic curve. The diagonal indicates no discrimination.

**Figure 2:**
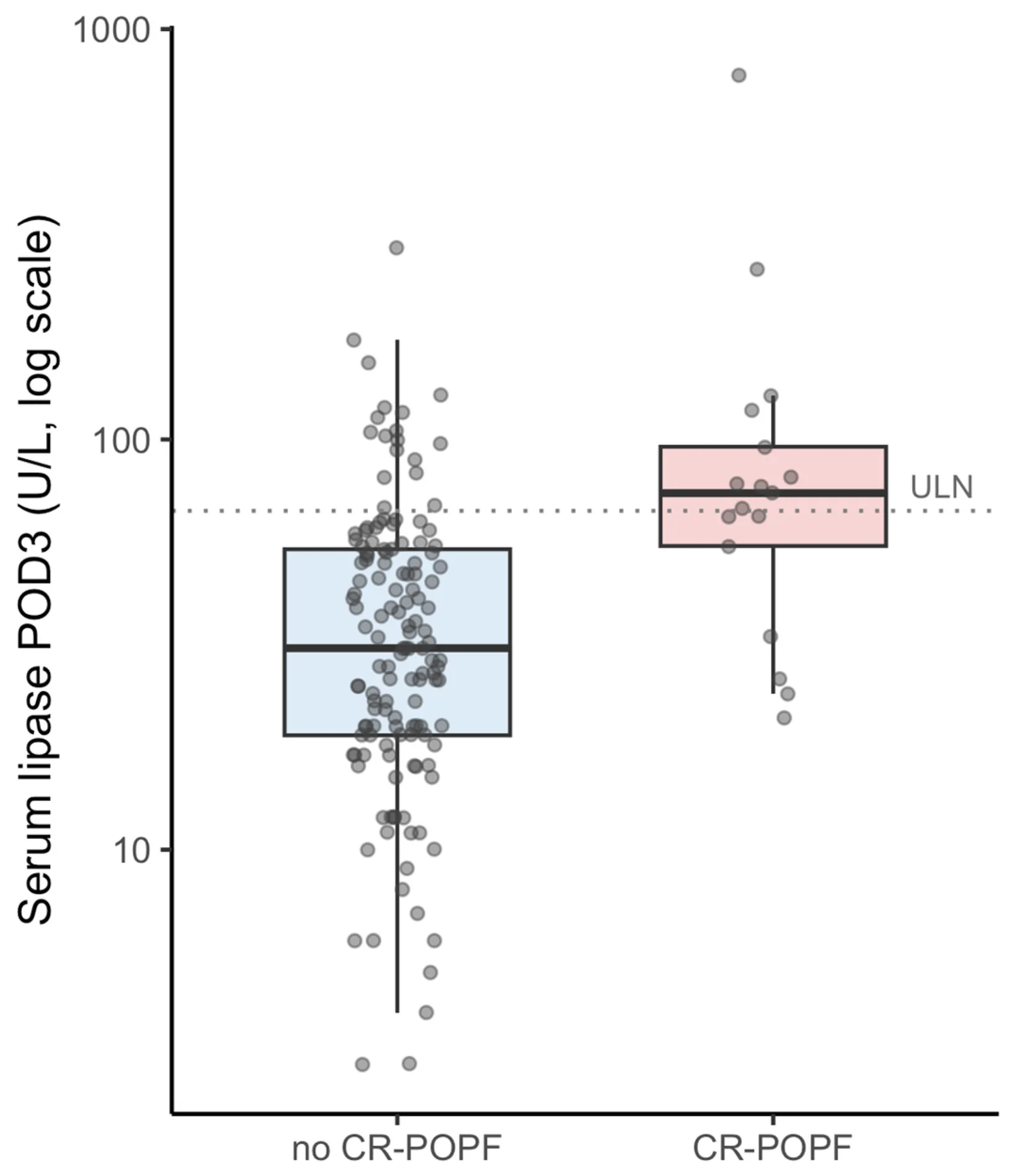
Postoperative day 3 (POD3) serum lipase according to clinically relevant postoperative pancreatic fistula (CR-POPF) status. Box plots show the median and interquartile range with individual observations (no CR-POPF, *n* = 140; CR-POPF, *n* = 17). The dashed horizontal line indicates the institutional upper limit of normal (ULN; 67 U/L). Values are shown on a logarithmic scale.

POD3 serum lipase was significantly higher in patients who developed CR-POPF than in those who did not: median 74 (IQR 55–96) versus 31 (IQR 19–54) U/L (*p* < 0.001; q = 0.001 after Benjamini–Hochberg correction). In univariable logistic regression, each doubling of POD3 serum lipase was associated with increased odds of CR-POPF (odds ratio 2.59, 95% CI 1.54 to 4.37; *p* < 0.001). The area under the receiver operating characteristic curve for POD3 serum lipase was 0.79 (95% CI 0.67 to 0.90); bootstrap optimism correction did not materially change the estimate (optimism-corrected AUC 0.79) (Table 2; Figure 1 and Figure 2). Among the ten laboratory markers evaluated, all three lipase-based variables remained significant after false discovery rate correction: POD3 serum lipase, peak lipase on POD1–3, and peak lipase on POD1–7 (q ≤ 0.005). POD3 C-reactive protein also remained significant after correction (q = 0.049), whereas the remaining six laboratory markers did not (Table 2). Because lipase and C-reactive protein were available in different subsets of patients, no formal paired comparison of their AUCs was performed.

### 3.4. Exploratory Analysis of Diagnostic Thresholds

At the prespecified institutional upper limit of normal (ULN, 67 U/L), the principal clinically interpretable threshold, sensitivity was 59% and specificity 87%, with a positive predictive value of 36%, a negative predictive value of 95%, a positive likelihood ratio of 4.6 and a negative likelihood ratio of 0.47 (Table 3). At the exploratory, data-derived Youden threshold of 64 U/L, nearly identical to the ULN, apparent sensitivity was 71% (95% CI 47 to 87; optimism-corrected 67%) and specificity 86% (95% CI 79 to 91), with a positive predictive value of 38%, a negative predictive value of 96%, LR+ 4.9 and LR− 0.34. At three times the ULN (201 U/L), specificity increased to 99% (positive predictive value 67%; positive likelihood ratio 16.5), but sensitivity decreased to 12%. Thus, lower thresholds were associated with high negative predictive value, whereas marked lipase elevation was highly specific but insensitive for CR-POPF. All estimates represent apparent performance in the derivation cohort. The negative predictive value depends strongly on CR-POPF prevalence in the analytic sample (approximately 11%) and should not be generalized to populations with a different prevalence.

### 3.5. Relationship with Fistula Severity

**Figure 3:**
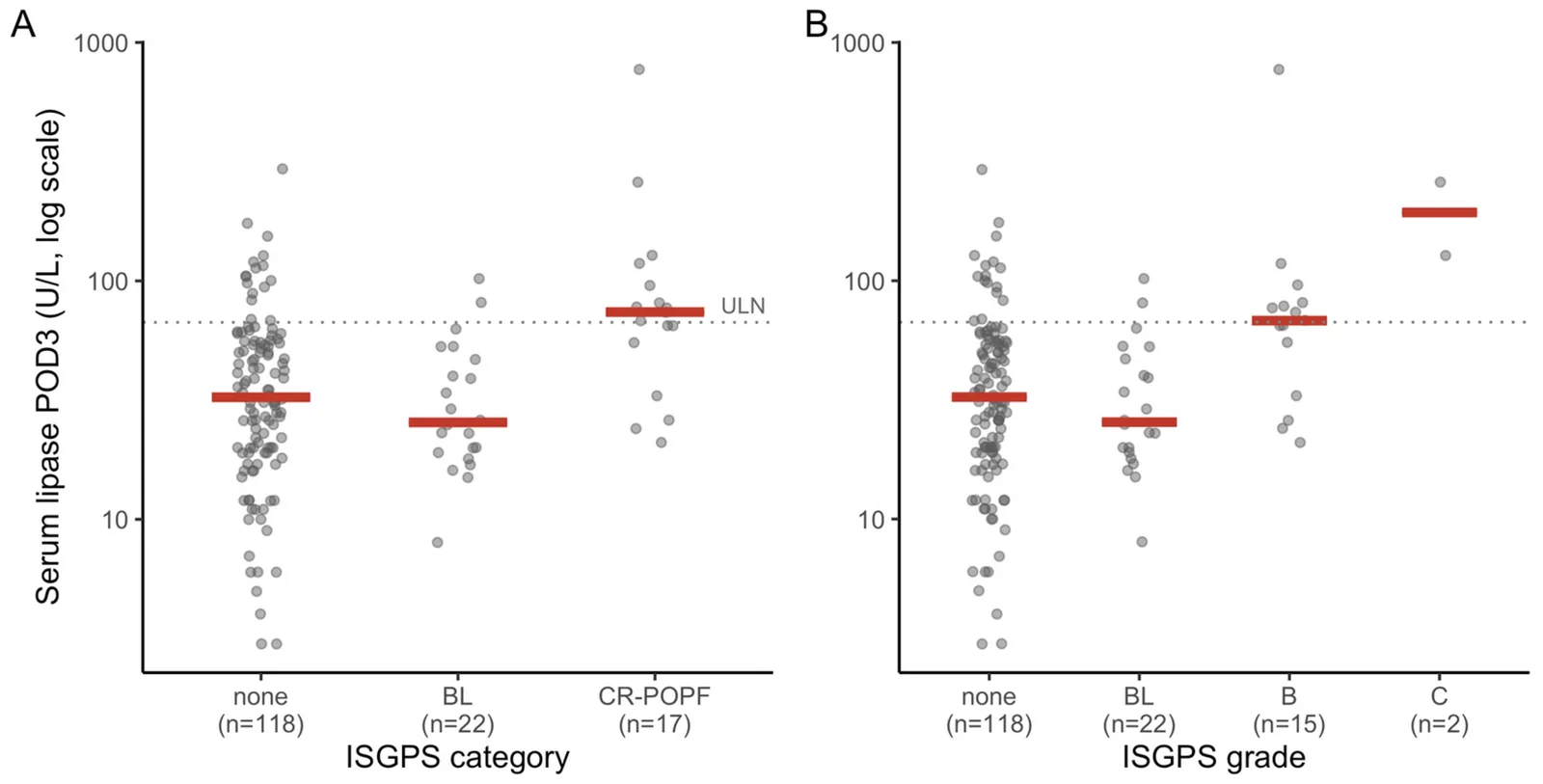
Postoperative day 3 (POD3) serum lipase across International Study Group on Pancreatic Surgery (ISGPS) categories (logarithmic scale; number of patients shown per category; *n* = 157 with an available measurement). (**A**) Inferential analysis with grades B and C combined into the clinically relevant category (no leak, *n* = 118; biochemical leak, BL, *n* = 22; CR-POPF, *n* = 17); Jonckheere–Terpstra trend test *p* = 0.012. (**B**) Descriptive display of all four grades (grade C, *n* = 2; exploratory). Individual observations with median bars; the dashed horizontal line indicates the institutional upper limit of normal (67 U/L).

POD3 serum lipase was analyzed across ISGPS categories in two complementary ways (Figure 3). In the inferential analysis, grades B and C were combined into the clinically relevant category, matching the study endpoint: median POD3 lipase was 32.5 U/L with no leak (*n* = 118), 25.5 U/L with biochemical leak (*n* = 22), and 74 U/L with CR-POPF (*n* = 17); the ordered association was significant (Jonckheere–Terpstra trend test *p* = 0.012; Kruskal–Wallis *p* < 0.001) and no category contained fewer than 17 observations. The descriptive display of all four grades (grade B: *n* = 15, median 68 U/L; grade C: *n* = 2, median 193.5 U/L) is shown in Figure 3B and is exploratory, given only two grade C observations with an available measurement. Notably, serum lipase was not elevated in biochemical leak compared with no leak (*p* = 0.47), indicating that the serum signal tracks clinically relevant fistula rather than any leak. In the subgroup of patients without an early POD1 lipase surge greater than 3 × ULN (*n* = 124, including 10 CR-POPF events), POD3 serum lipase remained higher in patients with CR-POPF than in those without CR-POPF (median 64 versus 29 U/L; *p* = 0.026). To characterize the temporal relationship between the POD3 measurement and the fistula, two time points were determined for each CR-POPF case: the first drain-fluid amylase elevation above three times the serum ULN at any postoperative time, and the first measurement satisfying the formal ISGPS biochemical criterion, which requires such an elevation on or after POD 3. An elevation was documented in 16 of 20 patients, with the first elevated value on POD 4 or later in 13 of 16 (median POD 5, range 0 to 26); the earliest elevations occurred in two fulminant grade C courses (POD 0 and POD 2) and one grade B case on POD 3. A measurement satisfying the formal temporal criterion was documented in 15 of 20 patients and occurred on POD 4 or later in 14 of 15 (median POD 6, range 3 to 26); of the two cases with earlier elevations, one subsequently met the formal criterion on POD 7, and the other (the patient who died on POD 3) had no drain-fluid measurement on or after POD 3 and was classified on clinical grounds. These dates reflect the timing of documentation rather than biological onset; the pathological process may already have been present by POD 3, and this analysis does not imply that POD3 serum lipase prospectively predicts the subsequent development of fistula.

### 3.6. Other Laboratory Markers

POD3 C-reactive protein was also higher in patients with CR-POPF than in those without (median 260 versus 217 mg/L); its AUC was numerically lower than that of POD3 serum lipase (AUC 0.67; *p* = 0.020; q = 0.049). Peak CRP showed only a borderline association after correction for multiple testing (q = 0.083). Peak serum amylase showed a weaker, non-significant association with CR-POPF (AUC 0.63; *p* = 0.082; q = 0.137). No significant association with CR-POPF was observed for POD3 leukocyte count (*p* = 0.41), POD1 hematocrit (*p* = 0.44), preoperative triglycerides (*p* = 0.61), or preoperative albumin (*p* = 0.38), nor for tumor type, tumor size, malignancy status, operative time, or diabetes.

### 3.7. Availability of Secondary Variables

Availability of secondary variables was incomplete (Table 4). Hematocrit on POD1, leukocyte count on POD3, and CRP on POD3 were available in more than 90% of patients, whereas POD3 serum lipase was available in 157 patients (78%). Preoperative albumin was available in 134 patients (66%), preoperative triglycerides in 117 patients (58%), drain amylase in 63 patients (31%), and pancreatic duct diameter in only 15 patients (7%). These missing data limited the assessment of some established risk factors and secondary laboratory markers, and should be considered when interpreting non-significant findings. Drain-fluid amylase was measured selectively, on clinical indication: it was available in 17 of 20 patients (85%) who developed CR-POPF versus 46 of 182 (25%) of those who did not. In the subset with both markers available (*n* = 56, including 15 CR-POPF events), the AUC was 0.95 for drain-fluid amylase and 0.76 for POD3 serum lipase (paired DeLong *p* = 0.019); because a drain-fluid amylase above three times the serum ULN is itself part of the ISGPS definition, its near-perfect discrimination is partly definitional. This comparison is descriptive only, given the selective, outcome-enriched sampling and the incorporation of drain-fluid amylase into the endpoint definition, and does not constitute evidence of comparative diagnostic performance. Among patients without CR-POPF, an elevated POD3 lipase (at or above the ULN) was not associated with a higher probability of drain-fluid sampling (33% versus 29%; *p* = 0.78), indicating that an isolated lipase elevation did not by itself trigger drain-fluid diagnostics.

## 4. Discussion

In this retrospective cohort of 202 patients undergoing distal pancreatectomy, POD3 serum lipase was associated with CR-POPF and showed moderate discrimination (AUC 0.79, 95% CI 0.67 to 0.90), with an OR of 2.59 per doubling of concentration and an ordered association across ISGPS severity grades. At a threshold close to the institutional ULN, the NPV was 96%; at 3 × ULN, specificity reached 99%. The purpose of this analysis was not to propose a stand-alone prediction score or to establish a new disease mechanism, but to clinically characterize a routine serum biochemical marker, not requiring drain-fluid sampling, at a decision-relevant postoperative time point.

The findings should be interpreted within an evidence base that already links postoperative pancreatic enzyme elevation to fistula. The European multicenter study by Perri et al. demonstrated that postoperative hyperamylasemia after distal pancreatectomy was associated with POPF occurrence and severity in 1192 patients [24]. Our results therefore do not show that postoperative enzyme elevation is a newly recognized phenomenon. Rather, they extend this concept to serum lipase measured specifically on POD3 after distal pancreatectomy. The association is biologically plausible because postoperative lipase elevation may reflect pancreatic injury, inflammation, or enzyme release; the present study cannot distinguish among these mechanisms. The persistence of the association among patients without an early POD1 lipase surge suggests that the POD3 signal is not explained solely by transient immediate postoperative hyperlipasemia.

Lipase-specific evidence has been stronger after pancreatoduodenectomy than after distal pancreatectomy. Chen et al. reported that POD1 serum lipase predicted CR-POPF after pancreatoduodenectomy [25], while other studies have evaluated postoperative pancreatic enzymes, drain-derived markers, and early risk models in related pancreatic-resection settings [26,27,28,29,30,31]. In a mixed cohort of pancreatic resections, Gasteiger et al. reported a POD3 serum lipase threshold of 51 U/L with an AUC of 0.799 for CR-POPF [32], which is close to the magnitude observed in our cohort. These observations support the biological plausibility of serum lipase as a marker of postoperative pancreatic injury, although its performance across resection types requires prospective external validation; the optimal timing and thresholds are likely procedure- and assay-specific.

Compared with CRP, which reflects systemic inflammation and has been associated with CR-POPF and postoperative collections [35,36,37], POD3 lipase showed numerically greater discrimination in our cohort (AUC 0.79 vs. 0.67). A formal paired comparison was not performed because the markers were not available in identical patient subsets. The two biomarkers may capture complementary biology: CRP reflects the systemic inflammatory response, whereas lipase more directly reflects pancreas-specific enzyme release. Dynamic postoperative risk-stratification approaches have also been described using serial biochemical information [38,39]. Future studies should evaluate whether combining a postoperative pancreatic enzyme marker with clinical, inflammatory, and drain-derived variables improves calibration and decision usefulness beyond any single marker.

Most established distal-pancreatectomy risk models rely on preoperative or intraoperative variables such as parenchymal thickness, texture, duct caliber, or technical factors [7,8,9,10,11,12,13]. A routine postoperative serum marker cannot replace those models, but it can provide dynamic information as the postoperative course evolves. This is particularly relevant when drain-fluid testing is unavailable or selectively performed. In our cohort, drain amylase was available in only 31% of patients, whereas POD3 serum lipase was available in 78%. The clinical value of serum lipase is therefore its accessibility and potential complementarity rather than superiority over the established drain-based definition of fistula. Because drains were placed routinely in this cohort, the present data do not address the value of serum lipase in a drainless pathway.

The threshold analyses should remain exploratory. A value near the ULN was associated with a high NPV, whereas marked elevation at 3 × ULN was highly specific but insensitive. These patterns may be clinically interpretable, but they do not justify using POD3 lipase alone to remove drains, trigger imaging, or determine discharge. Any future pathway should integrate serum lipase with symptoms, examination, drain findings when available, and established perioperative risk factors. External validation should use prespecified thresholds and prospectively standardized timing.

This study has several limitations. It was retrospective and single-center, and only 20 CR-POPF events occurred overall, with 17 events in the primary marker analysis. The small event count yielded wide confidence intervals and precluded stable multivariable adjustment. Established risk factors such as pancreatic thickness, texture, duct diameter, and body mass index were incompletely available; residual confounding therefore cannot be excluded. Consequently, independence of the lipase signal from established anatomical risk factors cannot be established with these data, and the observed association may in part reflect the same high-risk gland captured by those factors. Two of four grade C fistulas lacked a POD3 lipase measurement, raising the possibility of spectrum bias. The CR-POPF definition partly reflects management decisions that may themselves have been influenced by laboratory results (incorporation bias). In addition, outcome assessment was not blinded to laboratory values (review bias). The Youden-optimal threshold was derived and evaluated in the same dataset, so its apparent performance is optimistic despite bootstrap correction. Bootstrap correction constitutes internal validation only; it does not demonstrate reproducibility and cannot compensate for the low event count. Several laboratory and comorbidity variables were incompletely available and may have been missing not at random. The analytic platform for lipase was not centrally recorded across the ten-year study period, although the ULN remained stable. Operative practice also evolved over the decade (laparoscopic use 5% in 2016–2020 versus 29% in 2021–2026); although CR-POPF incidence and the lipase association were consistent across eras (Supplementary Table S1), temporal heterogeneity cannot be fully excluded. These limitations make the present findings hypothesis-generating and emphasize the need for prospective, multicenter validation.

The study also has strengths relevant to biomarker evaluation: it included consecutive distal pancreatectomies over a long observation period, used the standardized ISGPS endpoint, specified POD3 lipase as the primary marker before statistical analyses, reported marker availability transparently, controlled false discovery rate across exploratory laboratory comparisons, and used bootstrap internal validation. Together, these features support the hypothesis that POD3 serum lipase may be associated with CR-POPF, but they do not establish a validated clinical rule.

## 5. Conclusions

In this single-center retrospective cohort, postoperative day 3 serum lipase was associated with clinically relevant pancreatic fistula after distal pancreatectomy and showed exploratory discriminatory performance as a routinely available serum marker that does not require drain-fluid sampling. No clinically actionable cutoff can be recommended from these data: the observed thresholds were derived in this cohort and are not ready for implementation. Prospective multicenter validation with standardized serial sampling and prespecified thresholds is required before clinical use.

## Figures and Tables

**Table 1:** Patient characteristics and postoperative outcomes according to clinically relevant postoperative pancreatic fistula (CR-POPF). Length of stay is a postoperative outcome and is shown descriptively; it is not a baseline variable. Continuous variables are presented as median [interquartile range] and categorical variables as *n* (%). *p*-Values are from the Mann–Whitney U test for continuous variables and Fisher’s exact test for categorical variables and are descriptive. Hypertension and diabetes indicate documented diagnoses; documentation was incomplete, and percentages use the full group denominator.

Variable	CR-POPF (*n* = 20)	No CR-POPF (*n* = 182)	*p*
Age, years	70 [65–73]	65 [57–71]	0.025
Operative time, min	95 [60–130]	85 [65–120]	0.896
Length of stay, days	19 [14–34]	8 [7–10]	<0.001
Female sex, *n* (%)	11 (55)	122 (67)	0.323
Laparoscopic approach, *n* (%)	9 (45)	41 (23)	0.052
Hypertension (documented), *n* (%)	12 (60)	65 (36)	0.050
Diabetes (documented), *n* (%)	7 (35)	44 (24)	0.288
Malignancy, *n* (%)	6 (30)	35 (19)	0.251

**Table 2:** Laboratory markers according to CR-POPF status. Values are median [IQR]. AUC, area under the receiver operating characteristic curve; BH-FDR, Benjamini–Hochberg false discovery rate; CRP, C-reactive protein; POD, postoperative day. Raw *p*-values are from the Mann–Whitney U test; q-values are FDR-adjusted across the ten markers. *n* (CR/no) gives the available sample for each marker.

Marker	CR-POPF Median (IQR)	No CR-POPF Median (IQR)	AUC	*p*	q (BH-FDR)	*n* (CR/no)
Serum lipase POD3, U/L	74 [55–96]	31 [19–54]	0.79	<0.001	0.001	17/140
Peak lipase POD1–3, U/L	192 [93–271]	89 [52–152]	0.73	0.002	0.005	18/163
Peak lipase POD1–7, U/L	192 [109–271]	92 [54–152]	0.75	<0.001	0.002	18/167
CRP POD3, mg/L	260 [233–336]	217 [172–282]	0.67	0.020	0.049	18/167
Peak CRP, mg/L	289 [226–358]	237 [181–296]	0.64	0.042	0.083	20/176
Peak serum amylase, U/L	116 [98–174]	84 [54–146]	0.63	0.082	0.137	16/158
WBC POD3, ×109/L	17 [14–22]	16 [13–19]	0.56	0.412	0.492	20/173
Haematocrit POD1, %	35 [33–36]	34 [32–37]	0.55	0.443	0.492	20/176
Preop. triglycerides, mg/dL	92 [73–177]	91 [70–134]	0.55	0.612	0.612	12/105
Preop. albumin, g/L	44 [42–45]	44 [42–45]	0.43	0.383	0.492	17/117

**Table 3:** Apparent diagnostic performance of postoperative day 3 serum lipase for CR-POPF in the primary analytic sample (*n* = 157; 17 events). Sensitivity and specificity include 95% confidence intervals calculated using the Wilson method. PPV, positive predictive value; NPV, negative predictive value; ULN, upper limit of normal. The Youden threshold was selected and evaluated in the same dataset and requires external validation. LR+ and LR−, positive and negative likelihood ratios (added; less prevalence-dependent than PPV/NPV). Rows are ordered with the prespecified institutional ULN first; the data-derived Youden threshold is exploratory.

Threshold, U/L	Sensitivity % [95% CI]	Specificity % [95% CI]	PPV %	NPV %	LR+	LR−
≥67 (ULN)	59 [36–78]	87 [81–92]	36	95	4.6	0.47
≥64 (Youden, exploratory)	71 [47–87]	86 [79–91]	38	96	4.9	0.34
≥201 (3 × ULN)	12 [3–34]	99 [96–100]	67	90	16.5	0.89

**Table 4:** Availability of laboratory and clinical data in the cohort (N = 202). CRP, C-reactive protein; POD, postoperative day; WBC, white blood cell count.

Measurement	Available, *n* (%) (N = 202)
Haematocrit POD1	196 (97)
WBC POD3	193 (96)
CRP POD3	185 (92)
Preoperative glucose	182 (90)
Serum amylase	174 (86)
Serum lipase POD3	157 (78)
Histopathological diagnosis	137 (68)
Preoperative albumin	134 (66)
Preoperative triglycerides	117 (58)
Drain amylase	63 (31)
Pancreatic (Wirsung) duct diameter	15 (7)

## Data Availability

The participant-level data underlying this study are not publicly available because the ethics approval obtained for this study did not permit open deposition of patient records, and because the small clinical cohort may remain re-identifiable. A de-identified dataset and the analysis code are available from the corresponding author on request. Requests must include a brief research proposal and a data-security plan and are subject to institutional and Bioethics Committee approval and a data-use agreement; no authorship condition applies.

## Supplementary Materials

The following supporting information can be downloaded at: https://www.mdpi.com/article/10.3390/biomedicines14092020/s1. Supplementary File S1: STROBE checklist for cohort studies. Supplementary Table S1: Cohort characteristics, CR-POPF incidence and marker availability by study era (2016–2020 vs. 2021–2026).

## References

[1] Eshmuminov D., Schneider M.A., Tschuor C., Raptis D.A., Kambakamba P., Muller X., Lesurtel M., Clavien P.-A. (2018). Systematic review and meta-analysis of postoperative pancreatic fistula rates using the updated 2016 International Study Group Pancreatic Fistula definition in patients undergoing pancreatic resection with soft and hard pancreatic texture. HPB.

[2] Giuliani T., Siriwardena A.K., Vollmer C.M., Abu Hilal M., Adham M., Barreto S.G., Boggi U., Castillo C.F.-D., Del Chiaro M., Falconi M. (2026). The International Study Group for Pancreatic Surgery (ISGPS) definition and classification of postpancreatectomy mortality. Ann. Surg..

[3] Chui J.N., Sahni S., Samra J.S., Mittal A. (2023). Postoperative pancreatitis and pancreatic fistulae: A review of current evidence. HPB.

[4] Bassi C., Marchegiani G., Dervenis C., Sarr M., Abu Hilal M., Adham M., Allen P., Andersson R., Asbun H.J., Besselink M.G. (2017). The 2016 update of the International Study Group (ISGPS) definition and grading of postoperative pancreatic fistula: 11 years after. Surgery.

[5] Pulvirenti A., Ramera M., Bassi C. (2017). Modifications in the International Study Group for Pancreatic Surgery (ISGPS) definition of postoperative pancreatic fistula. Transl. Gastroenterol. Hepatol..

[6] Bonsdorff A., Sallinen V. (2023). Prediction of postoperative pancreatic fistula and pancreatitis after pancreatoduodenectomy or distal pancreatectomy: A review. Scand. J. Surg..

[7] Sugimoto M., Kendrick M.L., Farnell M.B., Nomura S., Takahashi N., Kobayashi T., Kobayashi S., Takahashi S., Konishi M., Gotohda N. (2020). Relationship between pancreatic thickness and staple height is relevant to the occurrence of pancreatic fistula after distal pancreatectomy. HPB.

[8] Schuh F., Mihaljevic A.L., Probst P., Trudeau M.T., Müller P.C., Marchegiani G., Besselink M.G., Uzunoglu F., Izbicki J.R., Falconi M. (2023). A simple classification of pancreatic duct size and texture predicts postoperative pancreatic fistula: A classification of the International Study Group of Pancreatic Surgery. Ann. Surg..

[9] Eshmuminov D., Karpovich I., Kapp J., Töpfer A., Endhardt K., Oberkofler C., Petrowsky H., Lenggenhager D., Tschuor C., Clavien P.-A. (2021). Pancreatic fistulas following distal pancreatectomy are unrelated to the texture quality of the pancreas. Langenbeck’s Arch. Surg..

[10] De Pastena M., van Bodegraven E.A., Mungroop T.H., Vissers F.L., Jones L.R., Marchegiani G., Balduzzi A., Klompmaker S., Paiella S., Rad S.T. (2023). Distal Pancreatectomy Fistula Risk Score (D-FRS): Development and international validation. Ann. Surg..

[11] Xu Y., Jin C., Fu D., Yang F. (2023). External validation of fistula risk scores for postoperative pancreatic fistula after distal pancreatectomy. Surgery.

[12] Zhu Y., Zhang W., Xue X., Qin L., Zou J., Tang Z. (2025). Developing and validating a nomogram to forecast clinically relevant postoperative pancreatic fistula following distal pancreatectomy. Gland Surg..

[13] Uemura S., Ban D., Esaki M., Nara S., Takamoto T., Mizui T., Shimada K. (2024). Risk factor analysis of postoperative pancreatic fistula after distal pancreatectomy with a focus on the peritoneum to portal vein distance on computed tomography. World J. Surg..

[14] Fukada M., Murase K., Higashi T., Yasufuku I., Sato Y., Tajima J.Y., Kiyama S., Tanaka Y., Okumura N., Takahashi T. (2023). Drain fluid and serum amylase concentration ratio is the most reliable indicator for predicting postoperative pancreatic fistula after distal pancreatectomy. BMC Surg..

[15] Zhu S., Yin M., Xu W., Lu C., Feng S., Xu C., Zhu J. (2024). Early drain removal versus routine drain removal after pancreaticoduodenectomy and/or distal pancreatectomy: A meta-analysis and systematic review. Dig. Dis. Sci..

[16] Yang F., Jin C., Hao S., Fu D. (2019). Drain contamination after distal pancreatectomy: Incidence, risk factors, and association with postoperative pancreatic fistula. J. Gastrointest. Surg..

[17] Connor S. (2016). Defining post-operative pancreatitis as a new pancreatic specific complication following pancreatic resection. HPB.

[18] Ikenaga N., Ohtsuka T., Nakata K., Watanabe Y., Mori Y., Nakamura M. (2021). Clinical significance of postoperative acute pancreatitis after pancreatoduodenectomy and distal pancreatectomy. Surgery.

[19] Andrianello S., Bannone E., Marchegiani G., Malleo G., Paiella S., Esposito A., Salvia R., Bassi C. (2021). Characterization of postoperative acute pancreatitis (POAP) after distal pancreatectomy. Surgery.

[20] Loos M., Strobel O., Mehrabi A., Mihaljevic A.L., Ramouz A., Dietrich M., Müller-Stich B.P., Diener M.K., Schneider M., Berchtold C. (2021). Postoperative acute pancreatitis is a serious but rare complication after distal pancreatectomy. HPB.

[21] Bannone E., Andrianello S., Marchegiani G., Malleo G., Paiella S., Salvia R., Bassi C. (2021). Postoperative hyperamylasemia (POH) and acute pancreatitis after pancreatoduodenectomy (POAP): State of the art and systematic review. Surgery.

[22] Romandini E., Perri G., Maekawa A., Bannone E., Sahakyan M.A., Kleive D.B., Ghorbani P., Marchegiani G., Montorsi R., Cattelani A. (2026). Postpancreatectomy acute pancreatitis after distal pancreatectomy: Tri-institutional international cohort. BJS Open.

[23] Poelsler L.H., Bellotti R., Pably D., Morell-Hofert D., Maier E., Cardini B., Oberhuber R., Resch T., Ponholzer F., Krendl F.J. (2026). Analysis of different post-operative hyperamylasemia criteria for defining post-pancreatectomy acute pancreatitis after distal pancreatectomy—A retrospective single-center study. J. Clin. Med..

[24] Perri G., Romandini E., Marchegiani G., Ghorbani P., Sahakyan M., Holmberg M., Cattelani A., Fretland Å., Montorsi R., Rodrigues I.D. (2025). Postoperative Hyperamylasemia (POH) Is an Early Predictor of Pancreatic Fistula Occurrence and Severity After Distal Pancreatectomy: Results from a European Multicentric Study. Ann. Surg..

[25] Chen H., Wang W., Zou S., Wang X., Ying X., Cheng D., Weng Y., Deng X., Shen B. (2022). Serum lipase on postoperative day one is a strong predictor of clinically relevant pancreatic fistula after pancreaticoduodenectomy: A retrospective cohort. Pancreatology.

[26] Mintziras I., Wächter S., Manoharan J., Albers M.B., Kanngiesser V., Maurer E., Bartsch D.K. (2023). Serum amylase on postoperative day 1 is superior to serum lipase in predicting clinically relevant pancreatic fistula after partial pancreaticoduodenectomy. Langenbeck’s Arch. Surg..

[27] Dalla Valle R., De Bellis M., Pedrazzi G., Lamecchi L., Bianchi G., Pellegrino C., Iaria M. (2015). Can early serum lipase measurement be routinely implemented to rule out clinically significant pancreatic fistula after pancreaticoduodenectomy?. Int. J. Surg..

[28] Paik K.Y., Oh J.S., Kim E.K. (2021). Amylase level after pancreaticoduodenectomy in predicting postoperative pancreatic fistula. Asian J. Surg..

[29] Doussot B., Doussot A., Ayav A., Santucci N., Deguelte S., Sow A.K., El Amrani M., Duvillard L., Piessen G., Girard E. (2024). Diagnostic accuracy of lipase as early predictor of postoperative pancreatic fistula: Results from the LIPADRAIN study. Ann. Surg. Open.

[30] Honselmann K.C., Antoine C., Frohneberg L., Deichmann S., Bolm L., Braun R., Lapshyn H., Petrova E., Keck T., Wellner U. (2021). A simple nomogram for early postoperative risk prediction of clinically relevant pancreatic fistula after pancreatoduodenectomy. Langenbeck’s Arch. Surg..

[31] Aghamaliyev U., Cepele G., Hofmann F.O., Knoblauch M., Kessler C., Crispin A., Weniger M., Andrassy J., Renz B.W., Werner J. (2024). Hyperlipasemia in the immediate postoperative period predicts postoperative pancreatic fistula after pancreatic resections. Surgery.

[32] Gasteiger S., Primavesi F., Göbel G., Braunwarth E., Cardini B., Maglione M., Sopper S., Öfner D., Stättner S. (2020). Early post-operative pancreatitis and systemic inflammatory response assessed by serum lipase and IL-6 predict pancreatic fistula. World J. Surg..

[33] Perri G., Bannone E., Frigerio I., Pellegrini R., Guerra M., Vella R., Cattelani A., Adinolfi E., Cillo U., Butturini G. (2026). Drain management after pancreatoduodenectomy: Risk-stratified dynamic algorithm. BJS Open.

[34] von Elm E., Altman D.G., Egger M., Pocock S.J., Gøtzsche P.C., Vandenbroucke J.P., STROBE Initiative (2007). The Strengthening the Reporting of Observational Studies in Epidemiology (STROBE) statement: Guidelines for reporting observational studies. Lancet.

[35] Sakamoto K., Ogawa K., Tamura K., Iwata M., Matsui T., Nishi Y., Nagaoka T., Funamizu N., Takai A., Takada Y. (2021). Postoperative elevation of C-reactive protein levels and high drain fluid amylase output are strong predictors of pancreatic fistulas after distal pancreatectomy. J. Hepatobiliary Pancreat. Sci..

[36] Baba K., Uemura K., Sumiyoshi T., Shintakuya R., Okada K., Harada T., Oka S., Ishii Y., Arihiro K., Murakami Y. (2026). Evaluating C-reactive protein in drainage fluid as a predictive biomarker for clinically relevant pancreatic fistulas following pancreaticoduodenectomy. Ann. Surg. Oncol..

[37] Lopez P., Pando E., Ortega-Torrecilla N., Puertolas N., Adell M., Fernandes N., Herms D., Barros M., Blanco L., Balsells J. (2024). The role of clinically relevant intra-abdominal collections after pancreaticoduodenectomy: Clinical impact and predictors. A retrospective analysis from a European tertiary centre. Langenbeck’s Arch. Surg..

[38] Kato T., Murase Y., Baba Y., Takase K., Watanabe Y., Okada K., Koyama I. (2025). Impact of dynamic risk stratification for postoperative pancreatic fistula using serum pancreatic amylase on the day after pancreatoduodenectomy. Surgery.

[39] Raza S.S., Nutu A., Powell-Brett S., Marchetti A., Perri G., Carvalheiro Boteon A., Hodson J., Chatzizacharias N., Dasari B.V., Isaac J. (2023). Early postoperative risk stratification in patients with pancreatic fistula after pancreaticoduodenectomy. Surgery.

