# Supplementary material for: Clinical Evaluation of Postoperative Day 3 Serum Lipase in Relation to Clinically Relevant Pancreatic Fistula After Distal Pancreatectomy: A Retrospective Cohort Study
Source: Biomedicines. 2026 Sep 8;14(9):2020. doi: 10.3390/biomedicines14092020 (PMC13604496; doi:10.3390/biomedicines14092020)
Supplement: Supplementary file 1 [file biomedicines-14-02020-s001.zip › File S1 - STROBE_Checklist_Biomedicines.pdf]

Supplementary File S1: STROBE Cohort-Study Checklist

Manuscript title: Clinical Evaluation of Postoperative Day 3 Serum Lipase in Relation to Clinically Relevant Pancreatic Fistula After Distal Pancreatectomy: A Retrospective Cohort Study

Authors: Oliwia Grząsiak-Kraj, Tomasz Kraj, Krzysztof Poznański, Agata Grochowska, Piotr Hogendorf, Adam Durczyński, and Janusz Strzelczyk

This checklist maps the manuscript to the STROBE recommendations for cohort studies.

Item	STROBE recommendation	Reported in manuscript
**1a**	Indicate the study design with a commonly used term in the title or abstract.	Title and Abstract (retrospective cohort study).
**1b**	Provide an informative and balanced summary of what was done and what was found.	Abstract.
**2**	Explain the scientific background and rationale for the investigation.	Section 1, Introduction.
**3**	State specific objectives, including any prespecified hypotheses.	Section 1, final paragraph.
**4**	Present key elements of the study design early in the paper.	Section 2.1, Study Design, Ethics, and Reporting.
**5**	Describe the setting, locations, and relevant dates, including recruitment, exposure, follow-up, and data collection periods.	Sections 2.1-2.3.
**6a**	Give eligibility criteria and the sources and methods of participant selection.	Section 2.2.
**6b**	For matched studies, give matching criteria and numbers of exposed and unexposed participants.	Not applicable; this was an unmatched cohort.
**7**	Clearly define outcomes, exposures, predictors, potential confounders, and effect modifiers; give diagnostic criteria where applicable.	Sections 2.4-2.5.
**8**	For each variable of interest, give data sources and details of measurement; describe comparability if more than one group exists.	Sections 2.3 and 2.5.
**9**	Describe efforts to address potential sources of bias.	Section 2.6 and Section 4 (limitations).
**10**	Explain how the study size was arrived at.	Section 2.6; all eligible cases during the study period, no formal sample-size calculation.
**11**	Explain how quantitative variables were handled and why any groupings were chosen.	Sections 2.5-2.6.
**12a**	Describe all statistical methods, including those used to control for confounding.	Section 2.6; rationale for not fitting a multivariable model is stated.
**12b**	Describe methods used to examine subgroups and interactions.	Sections 2.5-2.6 and 3.5.
**12c**	Explain how missing data were addressed.	Sections 2.6, 3.2, and 3.7; Table 4.
**12d**	If applicable, explain how loss to follow-up was addressed.	Section 2.2; outcomes assessed during index admission and documented 30-day follow-up.
**12e**	Describe any sensitivity analyses.	Section 2.6 and Section 3.5 (bootstrap validation and subgroup excluding early POD1 surge).
**13a**	Report numbers of individuals at each stage of the study.	Sections 3.1-3.2 (202 included; 157 in primary marker analysis).
**13b**	Give reasons for non-participation at each stage.	Section 2.2; reasons for missing POD3 lipase could not be determined retrospectively and are stated in Section 3.2.
**13c**	Consider use of a flow diagram.	Not included because counts screened and excluded by individual reason were not separately recorded; analytic sample availability is reported in Section 3.2 and Table 4.
**14a**	Give participant characteristics and information on exposures and potential confounders.	Section 3.1 and Table 1.
**14b**	Indicate the number of participants with missing data for each variable of interest.	Sections 3.2 and 3.7; Table 4.
**14c**	Summarize follow-up time.	Section 2.2; index admission and documented 30-day follow-up.

Item	STROBE recommendation	Reported in manuscript
**15**	Report numbers of outcome events or summary measures over time.	Section 3.1 (20/202 CR-POPF; 16 grade B and 4 grade C).
**16a**	Give unadjusted estimates and, if applicable, confounder-adjusted estimates with precision; state which confounders were adjusted for and why.	Section 3.3 (univariable OR with 95% CI); Section 2.6 and Discussion explain why no multivariable adjustment was fitted.
**16b**	Report category boundaries when continuous variables were categorized.	Sections 2.5-2.6 and Table 3.
**16c**	If relevant, translate estimates of relative risk into absolute risk for a meaningful time period.	Section 3.4 and Table 3 (PPV and NPV).
**17**	Report other analyses performed, such as subgroup and sensitivity analyses.	Sections 3.3-3.6; Table 2.
**18**	Summarize key results with reference to study objectives.	Section 4, opening paragraph.
**19**	Discuss limitations, including sources of potential bias or imprecision and their likely direction or magnitude.	Section 4, limitations paragraph.
**20**	Give a cautious overall interpretation considering objectives, limitations, multiplicity, and other evidence.	Section 4.
**21**	Discuss generalizability (external validity).	Section 4; prospective multicenter validation is recommended.
**22**	Give the source of funding and the role of funders.	Funding: no external funding.
