# Supplementary material for: Clinical Evaluation of Postoperative Day 3 Serum Lipase in Relation to Clinically Relevant Pancreatic Fistula After Distal Pancreatectomy: A Retrospective Cohort Study
Source: Biomedicines. 2026 Sep 8;14(9):2020. doi: 10.3390/biomedicines14092020 (PMC13604496; doi:10.3390/biomedicines14092020)
Supplement: Supplementary file 1 [file biomedicines-14-02020-s001.zip › Supplementary_Table_S1.pdf]

Supplementary Table S1. Cohort characteristics, CR-POPF incidence and marker availability by study era (2016–2020 vs. 2021–2026). CR-POPF, clinically relevant postoperative pancreatic fistula (ISGPS 2016 grade B/C); POD3, postoperative day 3.

Characteristic	2016–2020	2021–2026	p
Patients, n	37	165	—
CR-POPF, n (%)	2 (5.4)	18 (10.9)	0.54[1]
Laparoscopic approach, n (%)	2 (5)	48 (29)	—
POD3 serum lipase available, n (%)	21 (57)	136 (82)	0.002[1]

[1] Fisher's exact test.

In the contemporary era (2021–2026), which contained 16 of the 17 analysable CR-POPF events, POD3 serum lipase remained higher in patients with CR-POPF than in those without (median 71 vs. 31 U/L; $p < 0.001$, Mann–Whitney U test); the earlier era had too few events ($n = 1$ analysable) for a separate test.
